# A Set of Diverse Genes Influence the Frequency of White-Opaque Switching in *Candida albicans*

**DOI:** 10.1534/g3.120.401249

**Published:** 2020-06-02

**Authors:** Lucas R. Brenes, Matthew B. Lohse, Nairi Hartooni, Alexander D. Johnson

**Affiliations:** * Department of Microbiology and Immunology, and; ^†^ Department of Biochemistry and Biophysics, University of California, San Francisco, CA 94158

**Keywords:** Candida albicans white-opaque switching, non-transcriptional regulator genes, genetic screen

## Abstract

The fungal species *Candida albicans* is both a member of the human microbiome and a fungal pathogen. *C. albicans* undergoes several different morphological transitions, including one called white-opaque switching. Here, cells reversibly switch between two states, “white” and “opaque,” and each state is heritable through many cell generations. Each cell type has a distinct cellular and colony morphology and they differ in many other properties including mating, nutritional specialization, and interactions with the innate immune system. Previous genetic screens to gain insight into white-opaque switching have focused on certain classes of genes (for example transcriptional regulators or chromatin modifying enzymes). In this paper, we examined 172 deletion mutants covering a broad range of cell functions. We identified 28 deletion mutants with at least a fivefold effect on switching frequencies; these cover a wide variety of functions ranging from membrane sensors to kinases to proteins of unknown function. In agreement with previous reports, we found that components of the pheromone signaling cascade affect white-to-opaque switching; however, our results suggest that the major effect of Cek1 on white-opaque switching occurs through the cell wall damage response pathway. Most of the genes we identified have not been previously implicated in white-opaque switching and serve as entry points to understand new aspects of this morphological transition.

*Candida albicans* is an opportunistic fungal pathogen that is also a member of the human microbiome. When the immune system is compromised, *C. albicans* can cause systemic infections with fatality rates exceeding 40% (Kennedy and Volz 1985; Wey *et al.* 1988; Wenzel 1995; Calderone and Fonzi 2001; Kullberg and Oude Lashof 2002; Eggimann *et al.* 2003; Gudlaugsson *et al.* 2003; Pappas *et al.* 2004; Achkar and Fries 2010; Kumamoto 2011; Kim and Sudbery 2011). *C. albicans* is known for existing in several different morphological states. One such system is white-opaque switching, where *C. albicans* alternates between two cell types, named “white” and “opaque,” each with a distinct cellular and colony morphology (Figure 1A) (Slutsky *et al.* 1987; Soll *et al.* 1993; Johnson 2003; Pujol *et al.* 2004; Lohse and Johnson 2009; Soll 2009; Morschhäuser 2010; Porman *et al.* 2011). Roughly one-sixth of the transcriptome is differentially regulated between these two cell types (Lan *et al.* 2002; Tuch *et al.* 2010), and they also differ in metabolic preferences (Lan *et al.* 2002; Ene *et al.* 2016; Dalal *et al.* 2019), interactions with the innate immune system (Kvaal *et al.* 1997, 1999; Geiger *et al.* 2004; Lohse and Johnson 2008; Sasse *et al.* 2013; Takagi *et al.* 2019), responses to environmental cues (Si *et al.* 2013; Sun *et al.* 2015), and capacity to mate (Miller and Johnson 2002). The heritability of each cell type is a defining feature of white-opaque switching; in the absence of external signals switching between the two cell types occurs approximately once every 10^4^ cell divisions (Rikkerink *et al.* 1988; Bergen *et al.* 1990). Although rare under most conditions, switching is a reversible process that occurs without any chromosomal rearrangements or changes in genome sequence.

**Figure 1:**
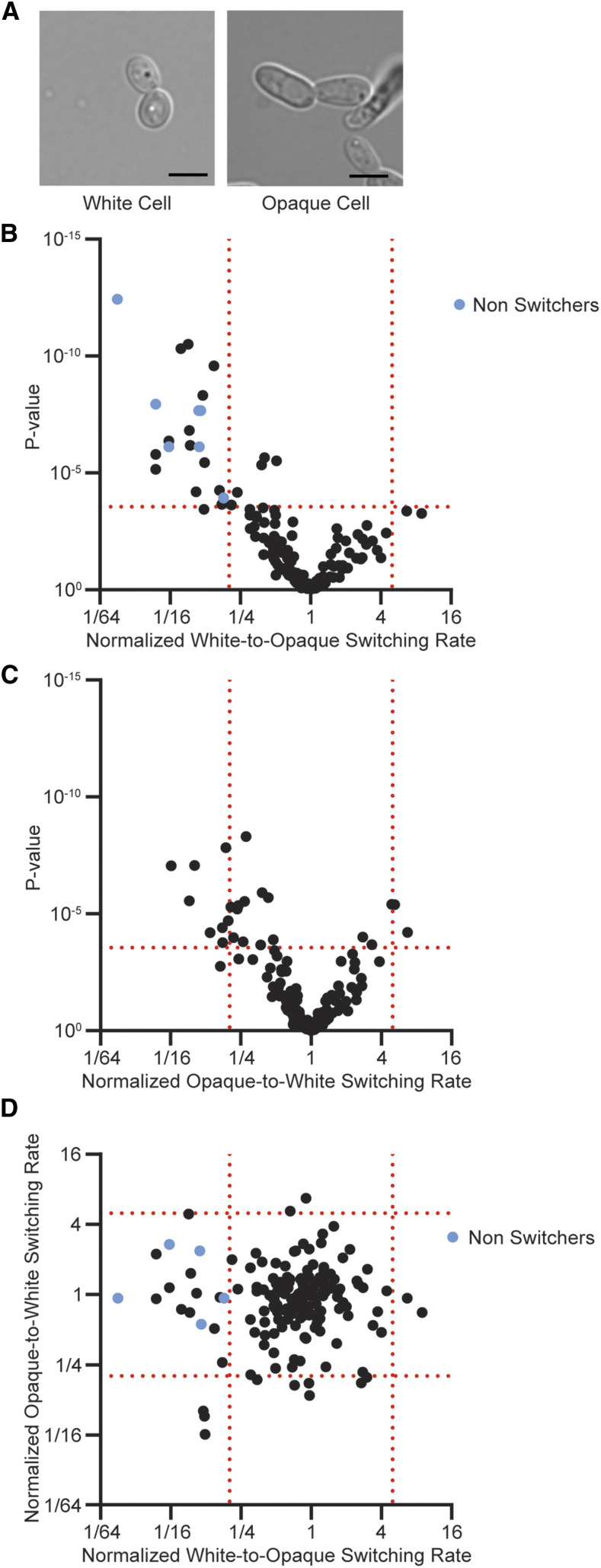
Identification of new genes that affect white-to-opaque or opaque-to-white switching. (A) Images of typical white (left) and opaque (right) *C. albicans* cells grown in liquid SCD+aa+Uri media at 25°C. Scale bar is 5µm, panel adapted from Lohse and Johnson 2016 (Lohse and Johnson 2016). (B-C) Volcano plots depicting the fold change in (B) white-to-opaque or (C) opaque-to-white switching rates. Vertical red lines indicate a fivefold change in switching rates. The horizonal red lines indicate α = 0.05 (Welch’s *t*-test with Bonferroni Correction for multiple comparisons, final thresholds of 2.91 × 10^−4^ and 2.94 × 10^−4^ respectively). The seven strains that did not switch from white-to-opaque in this assay are indicated in blue in panel B, we have set the switching frequency to 1 / (total number of colonies counted on all of the plates for that strain) for these strains to aid in visualization. The x-axes are plotted on a log2 scale, the y-axes are plotted on a log10 scale. (D) Comparison of normalized white-to-opaque and opaque-to-white switching rates for 170 genes for which we could test switching in both directions. A value of one represents switching at the wild type rate, values less than one reflect reduced switching, and values greater than one reflect increased switching. The five strains that did not switch in the white-to-opaque assay are indicated in blue, we have set the switching frequency to 1 / (total number of colonies counted on all of the plates for that strain) for these strains to aid in visualization. The x- and y-axes are plotted on a log2 scale.

Previous work identified a circuit with eight transcriptional regulators connected by interlocking feedback loops that regulates cell type switching and contributes to the stability of each cell type (Sonneborn *et al.* 1999; Srikantha *et al.* 2000, 2006; Huang *et al.* 2006; Zordan *et al.* 2006, 2007; Vinces and Kumamoto 2007; Wang *et al.* 2011; Hernday *et al.* 2013, 2016; Lohse *et al.* 2013; Lohse and Johnson 2016). A more recent systematic screen found that roughly twenty percent of transcriptional regulators tested (42 of 196) had at least fivefold effects on switching rates in one or both directions, suggesting that white-opaque switching is highly integrated with many aspects of *C. albicans*’ physiology (Lohse *et al.* 2016). Given this result, it is not surprising that genes from the cAMP/protein kinase A (Ramírez-Zavala *et al.* 2013; Ene *et al.* 2016; Cao *et al.* 2017; Ding *et al.* 2017), Hog1 osmotic/oxidative stress (Liang *et al.* 2014; Deng and Lin 2018), and CEK1 MAP kinase pathways (Ramírez-Zavala *et al.* 2013; Deng and Lin 2018) have been shown to affect switching rates. Likewise, several kinases (Ramírez-Zavala *et al.* 2013), GTPase associated proteins (Yang *et al.* 2018), chromatin modifying enzymes (Srikantha *et al.* 2001; Hnisz *et al.* 2009; Stevenson and Liu 2013), mediator subunits (Zhang *et al.* 2013), and DNA repair enzymes (Alby and Bennett 2009) have also been linked to alterations in switching rates.

Here, we report the construction of a deletion library of 172 diverse genes in a switching competent background. We determined the effects, if any, of these deletions on switching frequencies and found that roughly one-sixth of genes affected switching at least fivefold in one or both directions. We did not find any reliable predictors (*e.g.*, gene function or cell type expression patterns) for genes whose deletion would affect switching rates. We did find, in agreement with reports in the literature (Ramírez-Zavala *et al.* 2013; Deng and Lin 2018), that the Cek1 MAPK signaling cascade affects switching rates; however, our results suggest that Cek1 may be activated by a non-canonical pathway. Taken with previous observations, our results indicate that the frequency of white-opaque switching (which is dependent on the environment) is influenced by many inputs that extend across a surprisingly diverse set of cell processes.

## Materials and Methods

### Media and growth conditions

Unless otherwise noted, strains were grown on synthetic complete defined media containing yeast nitrogen base with 0.5% ammonium sulfate (6.7 g/L, BD #291940), amino acids (2 g/L), uridine (100 µg/mL), and 2% glucose (SCD+aa+Uri); plates also contained 2% agar. Recovery from glycerol stocks and plating assays were conducted at 25°. Secondary attempts to obtain opaque cells for the seven deletion strains that did not form opaque sectors during the initial switching assays were conducted at 25° on plates where 2% N-acetylglucosamine (GlcNAc, a monosaccharide glucose derivative that increases white-to-opaque switching rates) was substituted for glucose.

### Strain selection and construction

Lists of strains, plasmids, and oligonucleotides used in this study can be found in File S1. The deletion strains in this study were selected to encompass a wide range of GO term annotations (based on the data available at the Candida Genome Database (CGD, www.candidagenome.org)). Annotations of interest included functional groups (*e.g.*, kinases, phosphatases, transporters, proteases) and localization groups (*e.g.*, cell wall and/or surface). Additional strains were selected based on previous lists of genes that are differentially regulated at least twofold between the two cell types (Tuch *et al.* 2010) and/or whose transcript is transported in a She3-dependent manner (Elson *et al.* 2009). In total, 157 existing deletion strains were selected and fifteen new genes were selected for deletion. We note that this library does not represent an unbiased set of genes as a majority of the genes it contains were selected for study when the initial deletion strains were constructed, and then again when the existing deletion strains were selected for conversion. We also note three gaps in the numbering of strains in this library (26, 48, 164), these correspond to strains that were initially included in the library but later removed (*e.g.*, for being a putative transcriptional regulator).

The existing a/α deletion strains that comprise a majority of the strains used in this study (157 of 172) were taken from two previously reported deletion strain libraries (Elson *et al.* 2009; Noble *et al.* 2010). Given the inability of a/α cells of the SC5314 background to switch to the opaque cell type, we converted the existing a/α deletion strains to the switching competent **a**/Δ or α/Δ cell types through the random deletion of one copy of the MTL using the pJD1 construct and previously reported protocols (Lin *et al.* 2013; Lohse *et al.* 2016). Successful deletion of one copy of the MTL was confirmed by colony PCR against both the **a** and α *MTL* loci. Whenever possible, an a/Δ isolate was used (149 of 157 cases). Construction of the **a**/Δ wild type strain matched to this background has been previously reported (Hernday *et al.* 2013; Lohse *et al.* 2016); construction of the equivalent α/Δ wild type strain using pJD1 followed the same approach.

Fourteen of the fifteen new deletion strains were constructed in the SN152 (**a**/α *his1 leu2 arg4*) background using the *HIS1* and *LEU2* cassettes (Noble and Johnson 2005). Correct chromosomal integration, orientation of each marker, and loss of the ORF were verified by colony PCR. The same random MTL deletion approach was used to convert these strains, an **a**/Δ isolate was obtained for each strain.

Construction of the *opy2* deletion strain utilized the *SAT1* marker-based CRISPR protocol targeting *Candida maltosa LEU2* described by Nguyen and colleagues (Nguyen *et al.* 2017). We used a derivative of the hemizygous *LEU2* strain SN250 (itself a derivative of the aforementioned SN152 **a**/α *his1 leu2 arg4* strain with the *C. dubliniensis HIS1* and *C. maltosa LEU2* gene deletion cassettes integrated at the *C. albicans LEU2* locus) (Noble and Johnson 2005) which was converted to **a**/Δ by deleting the α copy of the MTL using pJD1 (Lin *et al.* 2013). The 90bp-annealed donor DNA (dDNA) contained homology to the regions directly upstream and downstream of the *OPY2* ORF. Each dDNA homology arm consisted of 44 bp and the two homology arms were separated by a two base pair GG insert added to create a potential gRNA site. Gene deletion was confirmed by colony PCR reactions verifying the loss of the *OPY2* ORF. After confirming gene deletion, the Cas9 ORF-gRNA-*SAT1* cassette was recycled by plating on Leu/His/Arg dropout plates and selecting for recombination events with an intact *CmLEU2* ORF. We selected against both leucine and histidine in order to avoid potential histidine auxotrophies arising during the recombination process (both *CmLEU2* and *CdHIS1* are present at the *CaLEU2* locus in the SN250-derived background).

### White-opaque switching assays

Large scale screening of white-to-opaque and opaque-to-white switching rates followed the protocol previously used for screening the transcription factor deletion library (Lohse *et al.* 2016), itself a refinement of a previously published protocol (Miller and Johnson 2002; Zordan *et al.* 2007). In brief, strains were allowed to recover from glycerol stocks for seven days on SCD+aa+Uri plates at 25°. After seven days, five colonies per strain that lacked visible sectors of the other cell type were resuspended in water and plated at a concentration of approximately 100 colonies/plate on six SCD+aa+Uri plates. Our experimental strategy was designed to monitor 600 colonies per strain; in practice we averaged 507 colonies per strain for white-to-opaque switching assays and 577 colonies per strain for opaque-to-white switching assays. At least four technical replicates of the wild type strain (24 plates in total) were included in each batch of switching assays. These plates were incubated for seven days at 25° before scoring colony phenotypes. Three phenotypes were noted: (1) the number of sectored colonies, (2) the number of fully switched colonies, and (3) the total number of colonies. The switching frequency was calculated as (1+2)/3. To account for any batch-to-batch variance, the switching frequencies for each strain were normalized to the average of the four wild type replicates from the same day. The **a**/Δ wild type was used as the control for **a**/Δ strains and the α/Δ wild type was used as the control for α/Δ strains. In the case of the seven strains that did not form any opaque sectors in this assay (*gpa2*, *hsl1*, *kex2*, *opy2*, *sld1*, *ssn3*, *tus1*), we have indicated the switching frequency as less than 1 / (total number of colonies counted on all of the plates for that strain) to aid in visualization. Unless otherwise noted, we use a fivefold effect on switching rates as a threshold for inclusion in subsequent analyses. Switching data for all 172 strains can be found in File S2. File S2 also contains the absolute switching rates and number of colonies counted for all deletion strains as well as the mean, standard deviation, and range of absolute switching rates for the wild type assays that were performed in parallel with each batch of strains.

### Switching assay statistical analysis

We used Welch’s *t*-test (two-tailed, unpaired, assuming unequal variance) to compare the switching rates for the individual mutant plates (usually six plates) to the switching rates for the individual plates from the four wild type controls (usually 24 plates) from the same day. We evaluated each mutant separately for the white-to-opaque and opaque-to-white assays. In order to correct for the multiple comparisons performed, the Bonferroni Correction with α = 0.05 was applied. All of the comparisons for a given type of assay (*e.g.*, all of the white-to-opaque experiments) were pooled for the multiple comparisons correction step, giving a number of hypotheses, m, of 172 for the white-to-opaque switching assays and of 170 for the opaque-to-white switching assays (for final thresholds of 2.91 × 10^−4^ and 2.94 × 10^−4^ respectively). At these thresholds, 20 of 21 strains with decreased white-to-opaque switching (including all seven strains that did not switch), eight of nine strains with decreased opaque-to-white switching, and both strains with increased opaque-to-white switching were significant. The *pcl5* (decreased white-to-opaque switching), *pkh2* and C3_00570C_A (increased white-to-opaque switching), and *spf1* (decreased opaque-to-white switching) fivefold effects were not statistically significant after the correction for multiple comparisons, although all four of these phenotypes would be considered significant if the less rigorous Benjamini-Hochberg procedure was used instead. Statistical testing of switching rates for all 172 strains can be found in File S2.

### Data availability

Strains and plasmids are available upon request. Supplemental Files have been uploaded to figshare. File S1 contains lists of strains, plasmids, and oligonucleotides used in this study. File S2 contains white-to-opaque and opaque-to-white switching frequencies for the 172 strains tested in this study. File S3 contains Supplemental Materials and Methods and Results relating to comparisons between white-to-opaque and opaque-to-white switching frequencies, GO terms, and genes whose transcripts are differentially regulated between cell types. File S4 contains data related to statistical testing for overrepresentation of genes affecting white-to-opaque or opaque-to-white switching in 42 GO-SLIM sets, She3 associated transcripts, two- or fourfold cell type enrichment, and genes with fivefold switching effects in the opposite direction. Supplemental material available at figshare: https://doi.org/10.25387/g3.12355049.

## Results

### Construction of an 174 member white-opaque switching competent gene deletion library

We previously reported the creation of an 196 member transcriptional regulator deletion library in which **a**/α mating type strains, which do not undergo white-opaque switching in the SC5314 background due to repression of Wor1 by the a1-α2 heterodimer, were converted to the **a**/Δ mating type (Lohse *et al.* 2016). Here, we used the same approach to convert 172 **a**/α deletion strains, a mixture of 157 existing deletion strains taken from two published libraries (Elson *et al.* 2009; Noble *et al.* 2010) and 15 newly created deletion strains, into the switching competent **a**/Δ or α/Δ backgrounds. In brief, we used a MTL locus deletion cassette to randomly delete either the **a** or the α mating type locus; whenever possible, we chose **a**/Δ strains rather than α/Δ strains for this screen. The deletion strains for conversion were selected based on criteria including gene localization or function (*e.g.*, cell wall proteins, kinases, phosphatases), differential transcriptional expression between white and opaque cell types, and association with specific groups of genes and/or pathways (“guilt by association,” *e.g.*, She3 mRNA transport targets, CEK1 signaling pathway). As such, we note that this library does not represent an unbiased set of genes; however, it does cover a wide range of gene types rather than a specific class of genes, an emphasis of previous screens (Hnisz *et al.* 2009; Ramírez-Zavala *et al.* 2013; Zhang *et al.* 2013; Lohse *et al.* 2016; Yang *et al.* 2018). A list of the 172 gene deletions strains can be found in File S1.

### Identification of 31 genes affecting white-opaque switching

We screened the 172 member switching capable gene deletion library for effects on white-to-opaque switching rates using an established plate based assay. In brief, we determined the fraction of colonies with one or more sectors and normalized this to the switching rate of the wild type control strain on the same day. We found that 23 gene deletions had at least fivefold effects on white-to-opaque switching relative to the wild type control (21 decreased switching, two increased switching; 13% of genes tested) (Figure 1B, Table 1, File S2). All but three of these results (decreased switching by *pcl5*, increased switching by *pkh2* and C3_00570C_A) met standard significance thresholds (Welch’s *t*-test with Bonferroni Correction for multiple comparisons, α = 0.05). Seven of the 21 reduced-switching gene deletions were severe (*gpa2*, *hsl1*, *kex2*, *opy2*, *sld1*, *ssn3*, *tus1*): no opaque sectors were observed across multiple plates, meaning that switching, if it occurred at all, must be extremely rare. When these seven strains were grown on media containing N-acetylglucosamine (GlcNAc, a monosaccharide glucose derivative that dramatically increases white-to-opaque switching rates), five of the seven produced opaque sectors indicating that these genes are not absolutely necessary for switching to or existing in the opaque state. We also note that the opaque versions of these five strains, once isolated, were stable on media with glucose as the carbon source suggesting that these genes did not affect maintenance of the opaque cell type. We did not observe any opaque sectors for the remaining two deletion strains, *hsl1* and *ssn3* (both kinases), under any conditions we tested. We do not know why deletion of these genes prevents white-to-opaque switching; however, both deletions increase filamentation (Wightman *et al.* 2004; Umeyama *et al.* 2005; Lu *et al.* 2019) and, given the many links between white-opaque switching and filamentation (Lohse *et al.* 2016), perhaps the increased tendency for filamentation competes with white-to-opaque switching. Alternatively, the genes may be required to maintain the opaque state.

**Table 1:** List of deletion strains with at least fivefold effects on white-to-opaque and/or opaque-to-white switching rates

Gene	Name	Normalized White-to-Opaque Frequency	White-to-Opaque P-value	Normalized Opaque-to-White Frequency	Opaque-to-White P-value
C1_14200W_A	C1_14200W_A	0.047	1.62E-06	0.918	8.43E-01
C3_00570C_A	C3_00570C_A	8.935	5.34E-04*	0.700	2.18E-01
C4_02720C_A	C4_02720C_A	0.165	5.55E-05	0.947	6.76E-01
CR_06450W_A	CR_06450W_A	0.148	2.63E-10	0.511	6.35E-04
C5_01680C_A	CCN1	0.119	4.75E-09	0.100	8.52E-08
C4_06480C_A	CEK1	0.089	3.13E-11	4.898	3.94E-06
C5_04130C_A	CHT2	3.005	1.15E-02	0.195	1.97E-05
C6_01070C_A	CIP1	2.695	4.75E-02	0.174	1.70E-04
C1_01270W_A	CTA9	0.077	4.78E-11	0.745	1.25E-01
C2_04050C_A	DCK1	0.104	6.33E-05	1.028	9.26E-01
C4_03470C_A	ECE1	0.345	7.54E-04	0.186	1.48E-08
C3_02240C_A	GPA2	< 0.114	2.14E-08	0.557	2.44E-03
C5_02840C_A	HSL1	< 0.047	1.15E-08	NA	NA
C4_03570W_A	HWP1	0.957	7.46E-01	0.173	3.91E-05
C2_09130C_A	IFF6	0.969	9.44E-01	0.136	6.49E-05
C1_08990C_A	KEX2	< 0.061	7.64E-07	2.700	5.75E-03
C2_01780W_A	MSB2	0.061	4.27E-07	1.147	1.82E-01
C4_00540C_A	NMD5	0.123	3.63E-06	0.063	8.88E-08
C1_08310W_A	OPY2	< 0.179	1.18E-04	0.929	3.53E-01
C5_05190W_A	PCL5	0.122	3.55E-04*	0.090	2.76E-06
C5_02720W_A	PEP12	0.663	2.20E-01	5.200	4.09E-06
C1_07230W_A	PHO15	0.905	7.41E-01	6.718	6.26E-05
C1_12410C_A	PKH2	6.675	4.31E-04*	0.930	6.72E-01
CR_02040W_A	PRK1	0.173	2.20E-04	0.261	1.58E-04
C1_09260C_A	PTC1	0.093	6.68E-07	1.520	1.75E-01
C1_09140C_A	SSU81	0.091	1.52E-07	0.700	5.45E-01
C1_04380W_A	SIT4	0.047	7.06E-06	2.223	1.40E-02
C3_02680C_A	SLD1	< 0.111	7.64E-07	2.371	1.21E-03
C2_06540C_A	SPF1	0.721	3.23E-01	0.166	1.74E-03*
C2_04260W_A	SSN3	< 0.110	2.14E-08	NA	NA
C1_04480C_A	TUS1	< 0.022	3.74E-13	0.928	7.95E-01

Switching frequencies are normalized to the average of four wild type switching assays performed on the same day. When no switching events were detected for a strain, the switching frequency is indicated as less than one divided by the number of colonies counted. Statistical significance is indicated (using Welch’s t-test with Bonferroni Correction for multiple comparisons, with α = 0.05, giving final thresholds of 2.91 x 10^−4^ for white-to-opaque switching and 2.94 x 10^−4^ for opaque-to-white switching). Genes with fivefold effects that did not meet these thresholds are indicated with an * after the relevant p-value. Absolute switching rates for each strain as well as wild type switching rates can be found in File S2.

We next identified deletion mutants that affected switching in the other direction, namely opaque-to-white switching. Of the 170 strains tested (we could not assess the *hsl1* and *ssn3* strains since they do not form opaque cells), eleven had fivefold or greater effects on opaque-to-white switching (nine decreased switching, two increased switching; 6% of genes tested) (Figure 1C, Table 1, File S2). All but one of these results (decreased switching by *spf1*) were significant by standard statistical criteria (Welch’s *t*-test with Bonferroni Correction for multiple comparisons, α = 0.05). Unlike the white-to-opaque results, none of these mutants completely blocked opaque-to-white switching. Between the two screens, eighteen percent of the genes tested (31 of 172) had a fivefold or greater effect on switching in at least one direction. To the best of our knowledge, only three of these 31 genes (*CEK1*, *GPA2*, *PHO15* (*PHO13*)) had previously been reported to affect white-opaque switching (Hnisz *et al.* 2009; Ramírez-Zavala *et al.* 2013; Ene *et al.* 2016; Deng and Lin 2018).

### Forward and backward switching rates are not linked

As was also observed in the transcriptional regulator screen, the effects of gene deletions on white-to-opaque and opaque-to-white switching are largely independent of each other: only three gene deletions (*ccn1*, *nmd5*, *pcl5*) had fivefold effects on both white-to-opaque and opaque-to-white switching (Figure 1D, Table 1, File S2). Given that a majority of genes affected switching in only one direction, it appears that the mechanisms and inputs for determining the frequencies of forward and reverse switching are largely independent of each other. Furthermore, we note that the three deletions with bidirectional effects decreased switching rates in both directions rather than having opposite effects. A mutation that simply destabilized the opaque cell would be predicted to decrease white-to-opaque switching and increase opaque-to-white switching. We do note that one of our deletion mutants did have this property: the *cek1* deletion reduces white-to-opaque switching eleven-fold and increases opaque-to-white switching 4.9-fold narrowly missing our fivefold threshold. This suggests that, rather than breaking switching in both directions like the *ccn1*, *nmd5*, and *pcl5* deletions, *CEK1* is important for both the establishment and the maintenance of the opaque cell type. However, the apparent scarcity of such mutants, coupled with the large number of mutants with effects in only one direction, suggests that forward and reverse switching frequencies have largely independent inputs.

### Gene function or expression patterns do not reliably predict white-opaque switching effects

Although our library of deletion mutants was biased toward genes we felt might impact white-opaque switching, the results indicate that no single criteria reliably predicts *a priori* whether a gene might affect white-opaque switching rates. In particular, differential mRNA expression in either white or opaque cells was not an accurate predictor, nor were intracellular location, GO term analysis, or even guilt by association (Files S3 and S4).

### Sho1, Msb2, and Opy2 activate Cek1 through a non-canonical route

Although most of the genes identified in this screen had not been previously implicated in white-opaque switching, several did coincide with previous reports. Activation of the Cek1 mitogen-activated protein kinase (MAPK) pathway, either through overexpression of an active form of the upstream kinase Ste11 or the downstream target of the pathway (the transcriptional regulator Cph1), has been reported to increase white-to-opaque switching rates (Ramírez-Zavala *et al.* 2013). To further explore this connection, our screen included deletions of three kinases known to act upstream of Cek1 (Cst20, Ste11, Hst7), the pheromone receptors Ste2 (for α factor) and Ste3 (for **a** factor), a component of the associated trimeric G-protein receptor (Cag1), and three proteins previously shown to activate the Cek1 pathway in response to cell wall damage (Msb2, Opy2, Sho1(Ssu81)) (Román *et al.* 2005, 2009; Herrero de Dios *et al.* 2013; Noble *et al.* 2017).

**Figure 2:**
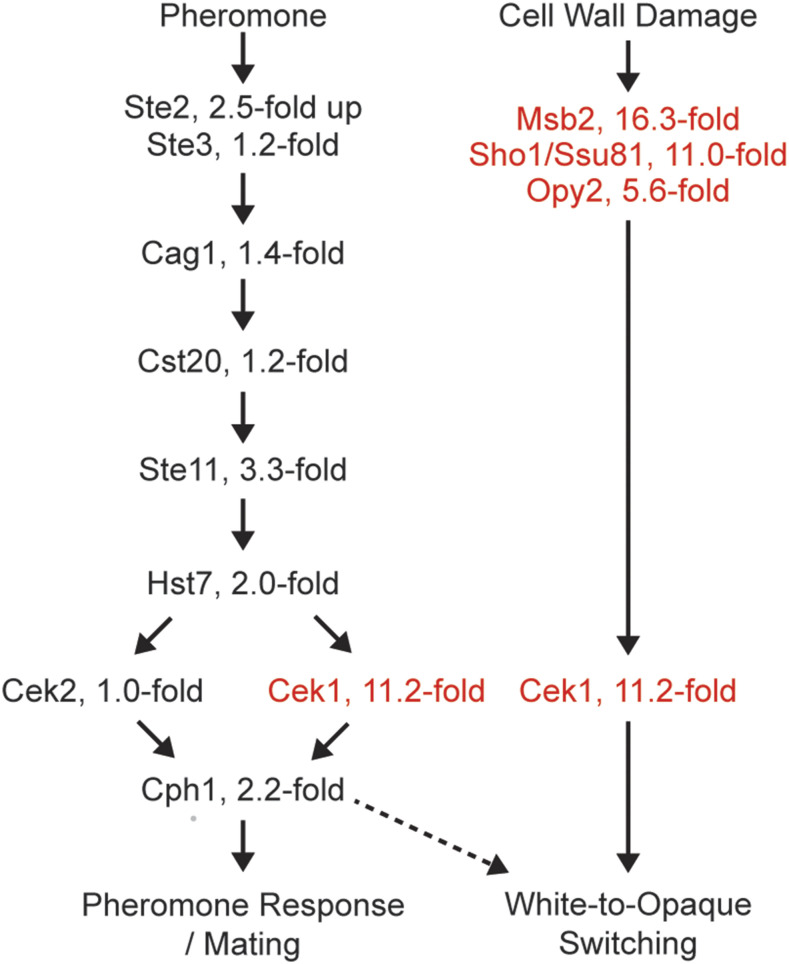
The pheromone and cell wall damage response pathways in *C. albicans* showing the effects of different deletions on white-to-opaque switching. Deletions with at least a fivefold decrease in white-to-opaque switching are indicated in red and deletions with smaller or undetectable effects are indicated in black. Data for the effects of the cph1 deletion on white-to-opaque switching are taken from a previous study (Lohse *et al.* 2016). These results suggest that, for white-opaque switching, the main input into Cek1 is through the cell wall damage response pathway, although there may be smaller contributions (indicated by the dashed line) from the pheromone response pathway.

Consistent with previous reports (Ramírez-Zavala *et al.* 2013; Deng and Lin 2018), we found that the *cek1* deletion (but not the *cek2* deletion) reduced white-to-opaque switching approximately ten-fold. However, deletion of the upstream MAP kinases of the MAPK pathway (Cst20, Ste11, Hst7), pheromone receptors (Ste2, Ste3), or trimeric G-protein receptor components (Cag1) had much smaller effects (at most threefold) on white-to-opaque switching. Deletion of Msb2, Opy2, and Sho1, on the other hand, resulted in six- to sixteen-fold reductions in white-to-opaque switching, similar in magnitude to the effect of the *cek1* deletion. Although it has been assumed that the signals from Cst20, Ste11, and Hst7 activate Cek1 for white-opaque switching though the MAPK kinase pathway, our results show that the signaling components of the cell wall damage pathway have more pronounced effects (Figure 2). These observations suggest that the major effects of Cek1 on white-opaque switching occur through the cell wall damage response pathway rather than the pheromone response pathway. Consistent with this idea, deletion of *CPH1*, a downstream effector of the pheromone response pathway, has only minimal effect (approximately twofold down) on white-to-opaque switching (Lohse *et al.* 2016).

## Discussion

Based on several criteria, including expression patterns, “guilt by association,” and hunches, we created a library of 172 *C. albicans* deletion strains (in a white-opaque switching background) and scored them for effects on white-to-opaque and on opaque-to-white switching rates. Although these deletion mutants do not reflect a random, unbiased selection, they do cover a more diverse set of genes than has been examined to date for white-opaque switching. We found that (1) roughly one-sixth of gene deletions had at least a fivefold effect on switching in either the forward or reverse direction, (2) the majority of the effects were uni-directional with very few deletions affecting switching in both directions, (3) decreases in switching rates were considerably more common than increases, and (4) no single criteria (*e.g.*, GO terms, “guilt by association,” or differential gene expression between white and opaque cells) had significant predictive value for genes whose deletion would affect switching rates. The fact that eighteen percent of genes tested affected white-opaque switching, coupled with the diversity of these genes, underscores the degree to which white-opaque switching is connected to many aspects of *C. albicans*’ cellular physiology. Consistent with this idea, 21% (42 of 196) of transcriptional regulator deletions had effects of similar magnitude (Lohse *et al.* 2016). The results of this and previous screens for deletion mutants and overexpression constructs that affect white-opaque switching rates suggest caution in concluding that a gene whose deletion causes a subtle increase or decrease in switching frequency is part of the core switching apparatus itself. Rather, the large number and variety of gene deletions that have these effects (but still allow cells to switch) likely affect inputs to the switch rather than the core switch itself.

Despite the aforementioned difficulty in predicting genes affecting white-opaque switching *a priori*, we note one instance of successful “guilt by association” where a gene with a known effect on switching, in this case Cek1, resulted in identification of additional genes (in this case *SHO1*, *MSB2*, and *OPY2*) with similar white-opaque switching phenotypes. Although genes in the canonical MAPK pathway were predicted to affect white-opaque switching to the same magnitude as Cek1, they did not and we note that our screen was useful in testing these predictions. Thus, although we were not particularly successful in predicting genes that affected white-opaque switching, the gene set we chose was useful in eliminating genes as well as identifying new genes that could serve as entry points for understanding other signals affecting white-opaque switching. Along this line, we note the interesting case of the white-enriched membrane protein C4_02720C_A and the opaque-enriched cell wall GPI-anchored protein Iff6, both of whose deletions decrease switching away from the cell type in which they are preferentially expressed; in other words, there are cell type regulated genes whose expression appears to decrease the stability of that cell type.

In conclusion, it is perhaps surprising that so many genes affect the frequency of white-opaque switching in *C. albicans*. This situation may arise, in part, because switching frequency is a delicate phenotype, but one that can be accurately quantified. For example, compared to the number of genes that affect switching rates, very few genes are essential for the switch itself. However, we do know that although switching itself is all-or-none, the frequency of switching can range continuously over several hundred-fold, depending on the environment (Dalal *et al.* 2019). The large number of genes identified thus far suggest that many different inputs determine the ultimate switching frequency of a given cell, yet we are far from understanding how this signal integration is performed and how results are transmitted to the switching apparatus.
